# Fast Transmitarray Synthesis with Far-Field and Near-Field Constraints

**DOI:** 10.3390/s22124355

**Published:** 2022-06-08

**Authors:** Susana Loredo, Enrique G. Plaza, Germán León

**Affiliations:** 1 Group of Signal Theory and Communications, Department of Electrical Engineering, University of Oviedo, 33204 Gijón, Spain; gleon@uniovi.es; 2 Department of Physics, University of Oviedo, 33204 Gijón, Spain; gonzalezenrique@uniovi.es

**Keywords:** transmitarray antennas, near-field synthesis, far-field synthesis, dielectric antennas, 3D printing

## Abstract

Millimeter-wave communications can potentially provide high-data-rate transmission. In addition, in the case of indoor small cells, new needs related to the radiation pattern of the antennas are emerging. In this work, a technique for the synthesis of planar transmitarray antennas with simultaneous near-field and far-field requirements is proposed. It is based on an iterative process, going from synthesized sources to generated field and back, through three operations: near-field computation as the sum of far-field contributions from the array elements, and inverse and direct fast Fourier transforms. As a result, the technique is very efficient from the point of view of computing time. In order to demonstrate the ability of the method, two examples are studied: one of them with a null in the near-field region and the other with a focal point, both pointing simultaneously in a specific far-field direction. The results are validated by manufacturing two dielectric “quasi-planar” prototypes at 26 GHz. The measure of the prototypes is in good agreement with the results advanced by the algorithm. These preliminary results suggest that the method can be extended to more complex scenarios.

## 1. Introduction

The fifth generation (5G) of mobile and wireless communication systems has brought about the emergence of new radio concepts and technologies (ultra-dense networks, ultra-reliable, massive machine communications, massive MIMO, etc.), as well as new and higher-frequency bands, where more bandwidth is available and, therefore, higher transmission rates can be achieved. The World Radiocommunication Conference held in 2019 (WRC-2019) identified several bands in the millimeter frequency range for International Mobile Telecommunications (IMT): 24.25–27.5 GHz, 37–43.5 GHz, 45.5–47 GHz, 47.2–48.2, and 66–71 GHz. Furthermore, United States led the world in making the 28 GHz band available for 5G, followed by several other countries, particularly Japan and South Korea, while most European Union countries have yet to auction off the 26 GHz or 28 GHz bands.

In this context, new and increasingly complex communication scenarios appear [1,2]. One case of interest is short-range millimeter wave communications for indoor small cells or femtocells, which could correspond to an office or meeting room. In such a scenario, there might be several devices and sensors closely distributed in the near field (NF) of the base station antennas, and different NF spots are, hence, required to provide coverage to the different devices [3,4], for transfer of information, power (wireless power transfer or WPT), or both (wireless power and information transfer or WPIT). Furthermore, it may also happen that some wireless devices are in the nearby regions of the base station antenna, while others are located in farther positions. Then, it will be necessary to simultaneously provide coverage in specific directions in regions more distant from the antenna, i.e., in the far-field (FF) region. In such complex scenarios, the requirements of the system would include then to provide simultaneous focused spots in the NF region and beams pointing at different directions in the FF region. Moreover, it might also be necessary to reduce the radiation at specific FF directions or NF positions to avoid or minimize interferences to or from other devices. Therefore, it becomes necessary to develop algorithms that allow synthesizing a radiation pattern with requirements in both the NF and the FF for indoor coverage. Power pattern synthesis techniques presented in [5,6] already included some NF constraints which were limited to the reduction in the radiated field level in certain areas close to the antenna. More recently, in [7], a technique was proposed for the synthesis of antenna arrays that can account simultaneously for NF focusing and FF specifications, with a view to future 5G scenarios and applications such as WPT or WPIT, where the devices of interest may be placed at both closer and farther positions.

At the same time, transmitarrays (TAs) have received much attention in the last decade [4,8,9,10,11,12,13]. They are planar array lenses characterized by their high efficiency and gain, low profile, and light weight, and they have attracted great interest as a feasible solution for beam steering and 5G multibeam antennas [13,14,15,16]. In parallel, also in recent years, 3D printing technology has emerged as a fast, low-cost, low-loss alternative for the fabrication of reflectarrays [17], lenses [18,19], and, in particular, TAs [4,11,20,21,22].

In this contribution, we propose a phase-only synthesis algorithm for TA antennas, which starts from given specifications in both the FF and the NF regions. This algorithm is the result of the combination of two independent FF [21,23] and NF [22] synthesis algorithms, which are mainly based on fast Fourier transform (FFT) and characterized by their short computing time. Furthermore, both algorithms were validated in previous studies [21,22] through the fabrication and measurement of 3D printed dielectric TAs. The result is a joint algorithm with similar characteristics: ease of implementation and fast execution. It was initially evaluated to generate a beam pointing at a given direction in FF, while simultaneously generating either a focusing spot or a null at a different direction in the region close to the antenna. Then, both TAs were manufactured using 3D printing technology and subsequently measured in a planar range and an anechoic chamber to determine both their NF and their FF radiation characteristics, with the measurements showing a good agreement with the model results. The size of the manufactured prototypes was limited by the available 3D printer, which allows a maximum manufacturing size of approximately 160 mm×160 mm.

## 2. Methodology

**Figure 1:**
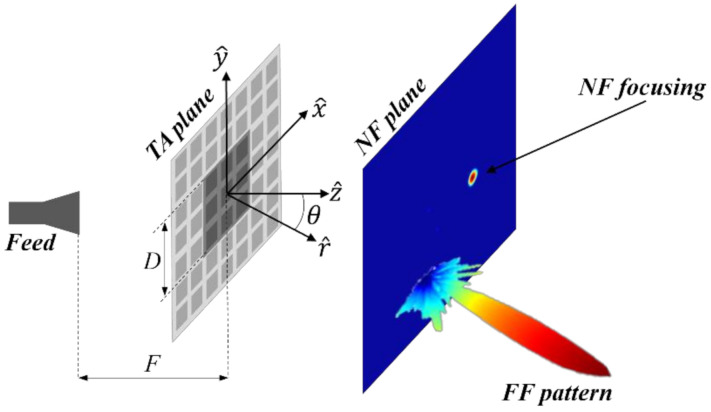
Transmitarray antenna with both NF and FF radiation characteristics.

In previous studies, two different algorithms were proposed to perform either FF [21,23] or NF [22] phase-only synthesis of planar array lenses or TAs, and they were validated by the design of pixelated quasi-planar dielectric lenses. Both proposals are based on a planar aperture model [24] and make use of FFT, a well-known, fast, and easy-to-implement tool, resulting in simple and short-computing-time algorithms. In this paper, we show that it is possible to combine both algorithms to model the radiation characteristics of a TA in terms of given requirements for both the NF and the FF radiation. Figure 1 illustrates this concept. The TA antenna is made up of the TA itself and the feed element, typically a horn antenna. Assuming a square TA, it consists of M×M square unit cells modeled as isotropic punctual sources. The field radiated by the horn (${\vec{E}}_{f}$) impinges both on these cells (darker elements in Figure 1) and on the spillover cells (lighter elements), i.e., a regular distribution of *virtual* cells around the TA, such that the total size of *TA plane* is $M_{s}\times M_{s}$ [22,23].

Denoting by ${\vec{E}}_{i}$ the electric field incident on *TA plane*, $E_{i}$ refers to the $\hat{x}/\hat{y}$ field component depending on the X/Y polarization of the feed, which can be written as

(1) $$E_{i}\left(x_{m},y_{n}\right)=\left|E_{f}^{mn}\right|e^{j\phi_{f}^{mn}},\;\;1\leq m,n\leq M_{s},$$

where $\left(x_{m},y_{n}\right)$ are the cartesian coordinates of a generic cell $\left(m,n\right)$ on the *TA plane*. This can be either a cell belonging to the TA or a spillover cell. Then, the field transmitted to the other side of the TA is obtained as

(2) $$E_{t}\left(x_{m},y_{n}\right)=\tau^{mn}\cdot E_{i}\left(x_{m},y_{n}\right)=\left\{\begin{array}{c} E_{i}\left(x_{m},y_{n}\right)e^{j\phi_{TA}^{\;mn}}\;\;\;\mathrm{for}\;\mathrm{TA}\;\mathrm{cells}\\ E_{i}\left(x_{m},y_{n}\right)\;\;\;\;\;\;\;\;\;\;\;\;\;\;\mathrm{for}\;\mathrm{SP}\;\mathrm{cells} \end{array},\right.$$

where $\tau^{mn}$ is the transmission coefficient, of unit magnitude and phase $\phi_{TA}^{\;mn}$, and SP cell refers to the *virtual* cells that model the spillover. For these cells, the transmission coefficient is always unity. The objective of the synthesis is to determine the matrix $\phi_{TA}$ of phases necessary to achieve the initial requirements.

To set a limit for the NF region, one can use the parameter $\gamma =r_{0}/\left(2D^{2}/\lambda \right)$, i.e., the focusing distance normalized to the FF region’s boundary. The focusing distance $r_{0}$ is defined from $\left(x=0,\;y=0,\;z=0\right)$ to the focusing spot. Better NF focusing is achieved for smaller values of γ [25]. On the contrary, NF focusing worsens when the value of γ increases. A typical value is γ=0.1. Then, for a frequency of 26 GHz, assuming that the TA size is D=150 mm, this would mean a focusing distance of 39 cm. This NF region can be extended by increasing the size of the TA. For example, if D=300 mm, with the same criteria, the distance would now be 1.6 m.

**Figure 2:**
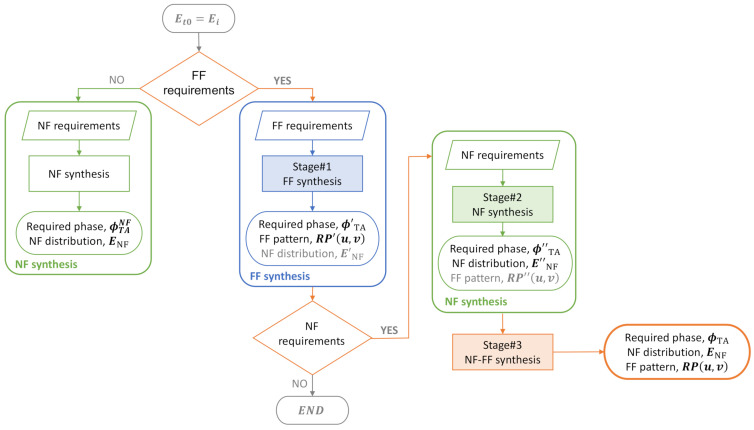
Flowchart of the general synthesis algorithm.

The flowchart of Figure 2 summarizes the whole set of solutions depending on the initial requirements: (1) only NF synthesis, if no FF constraints are imposed. This approach was used in [22] to synthesize a TA that focused the energy in two close points in different directions, as well as another TA with a flat-top beam also in the near region of the antenna. The latter was fabricated using 3D printing technologies, with the measured results showing very good agreement with those produced by the algorithm; (2) only FF synthesis, if no NF constraints are imposed. This algorithm was used in [21,23] to synthesize different radiation patterns (flat-top beam, isoflux, fan beam, and a two-beam pattern). It was also validated through the manufacturing and measurement of a dielectric prototype [21]; (3) both NF and FF synthesis, when constraints are imposed in both the near and the far region of the TA, which is the subject of this paper. As shown in this flowchart, to make the antenna radiation characteristics accommodate both FF and NF constraints, the proposed algorithm involves three stages:

1. Stage#1: only FF constraints are considered to perform the FF synthesis algorithm described in [21,23]. These constraints concern the pointing direction, beamwidth, and secondary lobe level (SLL). From these specifications, upper ($T_{up}^{FF}\left(u,v\right)$) and lower ($T_{low}^{FF}\left(u,v\right)$) auxiliary templates are defined.
  To initialize the algorithm, the TA is supposed to be transparent; hence, the initial electric field transmitted to the other side of the TA ($E_{t0}^{\#1}$) is the field incident from the feeding source ($E_{i}$), given by Equation (1).
  As a result of the synthesis, the matrix of the necessary phase shifts ($\phi^{\prime }{}_{TA}$) to be introduced by the TA in order to get a radiation pattern ($RP^{\prime }\left(u,v\right)$) that complies with the specifications (FF requirements) is obtained.
2. Stage#2: only NF constraints are considered to perform the NF synthesis algorithm described in [22] (there is a typo in Figure 2 of reference [22]: $E_{NF}$ *according to (4)* should read $E_{NF}$ *according to (3)*). NF requirements may include the position of the focus spot or the NF null, as appropriate, as well as the spot width and SLL. From these specifications, upper ($T_{up}^{NF}\left(x,y\right)$) and lower ($T_{low}^{NF}\left(x,y\right)$) auxiliary templates are defined.
  In this case, the starting point for the algorithm is the phases resulting from Stage#1. Therefore, the field initially transmitted to the other side of the TA is expressed as
  (3) $$E_{t0}^{\#2}\left(x_{m},y_{n}\right)=\left\{\begin{array}{c} E_{i}\left(x_{m},y_{n}\right)e^{j\phi \prime_{TA}^{\;mn}}\;\;\;\mathrm{for}\;\mathrm{TA}\;\mathrm{cells}\\ E_{i}\left(x_{m},y_{n}\right)\;\;\;\;\;\;\;\;\;\;\;\;\;\;\;\;\mathrm{for}\;\mathrm{SP}\;\mathrm{cells} \end{array}\right..$$
  As a result of the synthesis, the matrix of the necessary phase shifts (${\phi^{\prime \prime }}_{TA}$) to be introduced by the TA in order to get a field distribution on the near region (${E^{\prime \prime }}_{NF}$) that complies with the specifications (NF requirements) is obtained. With these new values of the transmission coefficient phase, the FF pattern becomes $RP^{\prime \prime }(u,v)$.
3. Stage#3: the above two constraints are merged to take into account both the NF and FF constrains.
  The starting point for the algorithm is now the phases resulting from Stage#2. Therefore, the field initially transmitted to the other side of the TA ($E_{t0}^{\#3}$) is expressed as shown in Equation (3), changing $\phi_{TA}^{\prime mn}$ to $\phi_{TA}^{\prime \prime mn}$.
  As a result of the synthesis, the matrix of the necessary phase shifts ($\phi_{TA}$) to be introduced by the TA in order to get a field distribution in the near region ($E_{NF}$) that complies with NF constraints and a radiation pattern ($RP\left(u,v\right)$) that complies with FF constraints is obtained.

**Figure 3:**
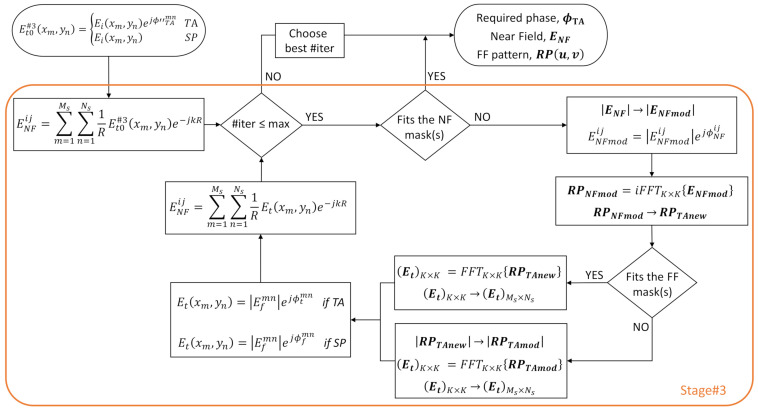
Flowchart of Stage#3: NF–FF synthesis.

Figure 3 shows the flowchart of the algorithm proposed for Stage#3. From the initial transmitted field, the near field on the plane $z=z_{0}$ is estimated for each position $\left(x_{i},y_{j}\right)$ as the sum of the FF contributions of both the TA elements and the spillover elements. Since we are assuming square TAs, $N_{s}=M_{s}$. Then, the NF amplitude is compared to the NF defined templates [22], giving place to a modified near field ($E_{NFmod}$).

To achieve this new NF distribution, a new transmitted field on the TA is required, resulting in a new FF radiation pattern ($RP_{TAnew}$), obtained as already exposed in [22].

Lastly, prior to retrieve the necessary transmitted field, this radiation pattern must be compared to the FF templates and trimmed if necessary to the restrictions imposed by them, giving place in that case to a modified radiation pattern ($RP_{TAmod}$).

(4) $$\left|RP_{TAmod}\left(u,v\right)\right|=\left\{\begin{array}{c} \frac{1}{\sqrt{2}}T_{up}^{FF}\left(u,v\right),\left|RP_{TAnew}\left(u,v\right)\right|>T_{up}^{FF}\left(u,v\right)\\ \sqrt{T_{low}^{FF}\left(u,v\right)},\left|RP_{TAnew}\left(u,v\right)\right|<T_{low}^{FF}\left(u,v\right)\\ \left|RP_{TAnew}\left(u,v\right)\right|,\;\;\;\;\;\;\;\;\;\;\;\;\;\;\;\;\;\;\;\;\;\;\;\;otherwise. \end{array}\right.$$

Applying now a 2D FFT, the necessary transmitted field ($E_{t}$) is finally retrieved. It is a K×K matrix, from which extra elements must be removed to fit its size to that of the TA, including the spillover elements. Then, the amplitude of the transmitted field is restored to that of the incident field, as well as the phase of the spillover elements, since no synthesis can be applied to these *virtual* elements. Moreover, the near field due to this new transmitted field is obtained, repeating the process iteratively until the maximum number of iterations is reached. From the set of solutions, the “best option” is chosen on the basis of different criteria: percentage of positions/directions that do not fulfill the templates, mean error, and visual inspection. This process is more clearly illustrated with the cases analyzed in Section 3.

Finally, the required phases on the TA are obtained as

(5) $$\phi_{TA}^{\;mn}=\phi_{t}^{\;mn}-\phi_{f}^{\;mn}.$$

## 3. Results of the Synthesis Algorithm and Experimental Validation

Two different cases are presented to illustrate and validate the proposed NF–FF synthesis methodology. The operating frequency was 26 GHz in both cases, and the periodicity of the cells was 0.3λ×0.3λ. The side of the TA, therefore, had a length D=0.3λ·M, and $M_{s}=2M$. The feeding element was placed at a distance F=80 mm from the TA in the *z*-axis, pointing at its center (see Figure 1). The same horn used in previous prototypes [21,22] was used, whose gain at 26 GHz was around 15 dB. This horn was designed by the authors and manufactured using 3D printing techniques.

To initialize the algorithm, it is necessary to know the field incident from the horn on *TA plane*. In previous studies, two different approaches were considered for this: either modeling the horn using a $cos^{q}\theta$ model [23] or using the NFPC (Near-Field Plane Cuts) model [21,22] that was previously presented in [24]. In this work, the field generated by the horn on the plane of the TA was measured in the same planar range facility later used to measure the manufactured prototypes. The horn field was captured in an area large enough to include also the positions associated with the spillover cells, and then introduced into the algorithm.

### 3.1. Case 1: Far-Field Beam and Null in Near Field

The objective of this first case was to design a TA with the following constraints:

- FF: pointing at $\left(\theta =-20{}^{\circ},\;\phi =0{}^{\circ}\right)$ with SLL lower than −15 dB outside the main lobe.
- NF: null at the position $\left[D\cdot tg\theta^{\prime },0,D\right]$, with $\theta^{\prime }=20{}^{\circ}$, and level lower than −10 dB for all other directions except that of the FF beam.

**Figure 4:**
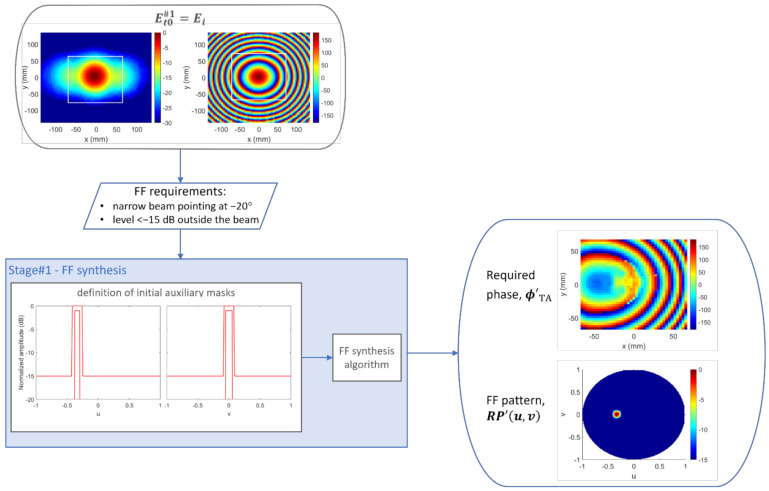
Case 1, Stage#1: inputs and outputs. Field magnitudes are presented normalized in dB, and the phases are presented in degrees.

A TA of 40×40 cells, i.e., D=138.5 mm at the operating frequency, was proven to be large enough to satisfy this objective, while the TA + spillover set consisted of 80×80 elements. The first step, as illustrated in Figure 2, was to perform the synthesis with only FF requirements in consideration. Figure 4 illustrates both inputs and outputs of Stage#1. The field measured for the horn ($E_{i}$) is shown in both amplitude (left) and phase (right), with the white line indicating the limit between TA and spillover areas. From the FF specifications, auxiliary templates were defined, since the FF synthesis algorithm [21,23] is based on the use of upper and lower templates. The ultimate goal, in this case, was not strict compliance with the masks, but rather a means to achieve agreement with the initial specifications, which were not as restrictive. The templates were defined in the whole UV grid, although only the main cuts are shown in Figure 4.

**Figure 5:**
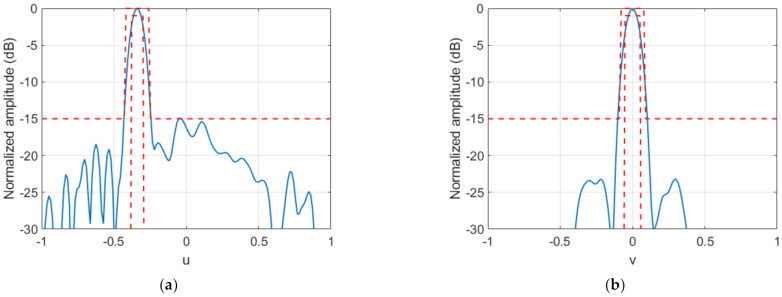
Case 1, Stage#1: main cuts of the synthesized radiation pattern and comparison with templates. (**a**) cut v=0; (**b**) cut u=−0.3386.

**Figure 6:**
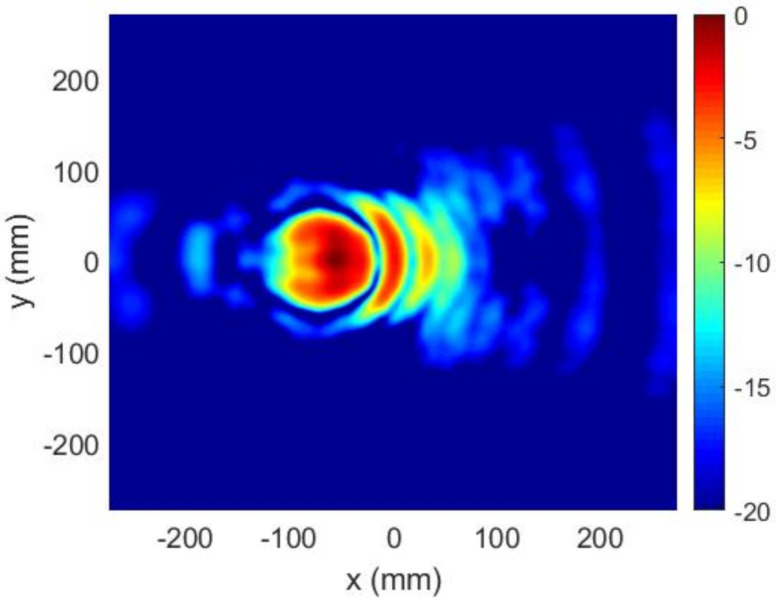
Case 1, Stage#1: near field (${E^{\prime }}_{NF}$) at z=D (normalized amplitude, dB).

Regarding the FF synthesis algorithm, the maximum number of iterations was set to 100, implying a computation time of about 1.5 s in a laptop with an Intel Core i5-5200U CPU at 2.2 GHz. The resulting FF pattern, as well as the required transmission phase on the TA, is shown in Figure 4. After this, the percentage of directions not fulfilling the templates was 0.42%. Figure 5 shows how these nonconformities were derived from the self-imposed templates, but that the radiation pattern obtained nevertheless conformed perfectly to the starting specifications: the maximum was at $\left(u=-0.3386,\;v=0\right)$, i.e., $\left(\theta =-19.79{}^{\circ},\;\phi =0{}^{\circ}\right)$, and only 0.0126% directions outside of the beam presented a level above −15 dB, with a mean error of 0.07 dB. The error of a noncompliant direction was defined as the difference in dB between the field value at that direction and the desired value given by the template. The solution provided by the FF synthesis for the transmission phase led to the NF distribution shown in Figure 6 on the plane z=D=138.5 mm.

**Figure 7:**
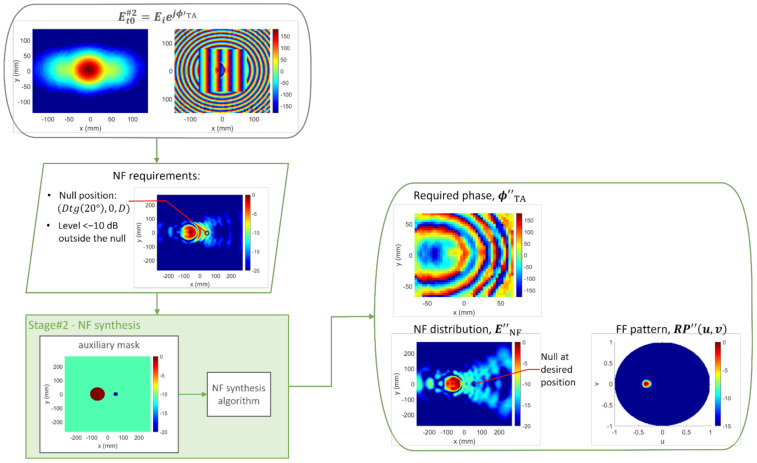
Case 1, Stage#2: inputs and outputs. Field magnitudes are presented normalized in dB, and the phases are presented in degrees.

Next, the NF synthesis algorithm was applied, considering only NF requirements. Figure 7 illustrates both inputs and outputs of Stage#2. On the basis of the specifications, an auxiliary template was set. In this case, a unique template was needed to define the location of the null, located around $\left(x=50.41\;\mathrm{mm},\;y=0\right)$. The NF grid consisted of 100,489 points (317 × 317) sampled every 0.15λ.

**Figure 8:**
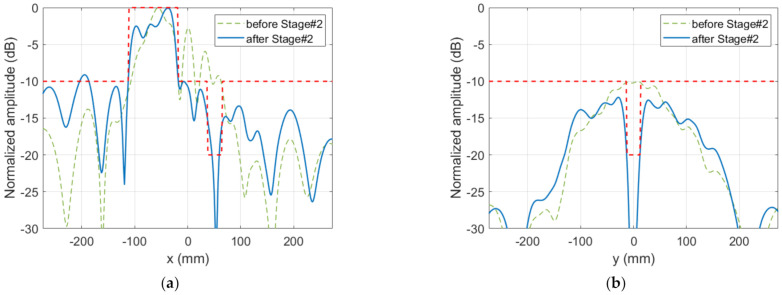
Case 1, Stage#2: main cuts of the synthesized near field and comparison with templates. (**a**) cut y=0; (**b**) cut x=51.9 mm.

**Figure 9:**
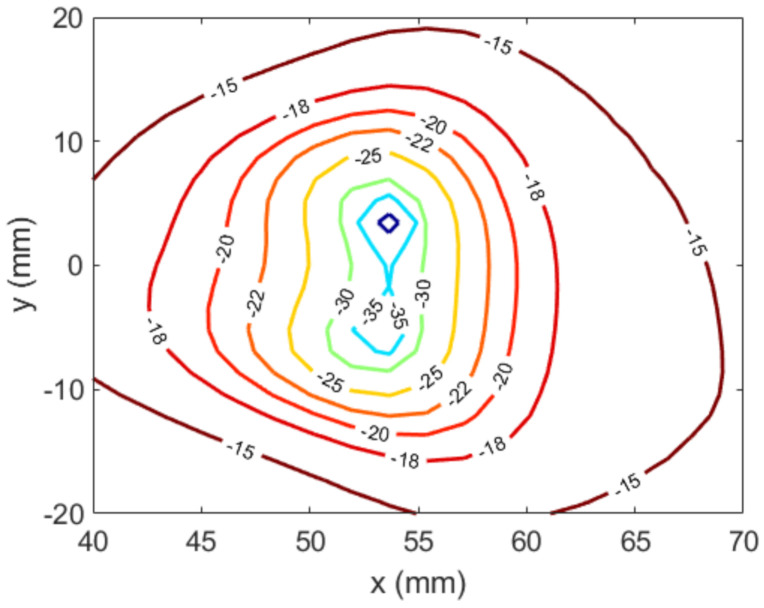
Case 1, Stage#2: near field (${E^{\prime \prime }}_{NF}$) around the desired null position (normalized amplitude, dB).

The maximum number of iterations was set to five. To choose the best iteration, two criteria were taken into account: the percentage of locations not fulfilling the template(s) and the mean error at those noncompliant positions. An iterative process was applied, such that, in each iteration, the two worst cases were eliminated according to each criterion. After this process, for which a time of 70 s was invested in the abovementioned computer, the best result was found after the second iteration. In that case, the percentage of noncompliant positions was 0.42%, and the mean error was 1.06 dB. The same considerations discussed above regarding mask compliance were taken into account. Moreover, the NF results, as shown in Figure 8 and Figure 9, agreed well with the specified constraints. The percentage of positions whose level was higher than −10 dB instead of lower was 0.32%, and the mean error at those positions was 0.52 dB.

Figure 7 also shows the new required transmission phase ${\phi^{\prime \prime }}_{TA}$ at the TA, as well as the FF pattern obtained with this new phase distribution, which hardly changed with respect to that obtained in Stage#1. The percentage of directions outside of the beam whose level was above −15 dB became 0.021%, while the mean error increased slightly.

Next, Stage#3 was executed, taking into account both NF and FF constraints. A maximum of five iterations were run, and the best choice turned out to be the second one. Table 1 summarizes the whole process in terms of noncompliance of the templates and computation times. At this point, it is worth remembering that the algorithm does not find an optimal solution. Furthermore, error data are referred to templates, which are the auxiliary tools to satisfy the given specifications; hence, the visual inspection of results can sometimes also be a useful tool for understanding error data. For example, cases of noncompliance often occur at the transition between high and low template levels; however, this is not relevant for compliance with the specifications. Given the immediacy of the proposed method, which is its great advantage together with its relative easiness, different solutions can be studied, e.g., defining different auxiliary templates or different number of cells, and both error data and visual results can be analyzed.

Turning back to the results of Table 1, the execution of Stage#3 slightly improved the results of the FF pattern but worsened the NF results somewhat in comparison to Stage#2. Either solution was good enough for our purpose, and the one resulting from Stage#2 was eventually chosen to manufacture the prototype.

As in previous studies [21,22], the algorithm was validated by the manufacture and measurement of a dielectric TA, where the unit cell was a dielectric slab and phase tuning was achieved by varying the slab height in the different cells that make up the array. This technology was chosen because of its low cost and ease of manufacture, since a general-purpose 3D printer can be used.

**Figure 10:**
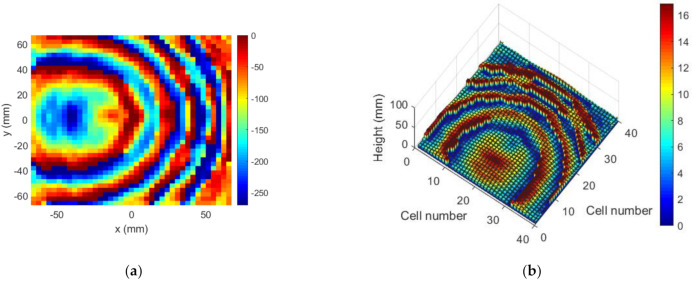

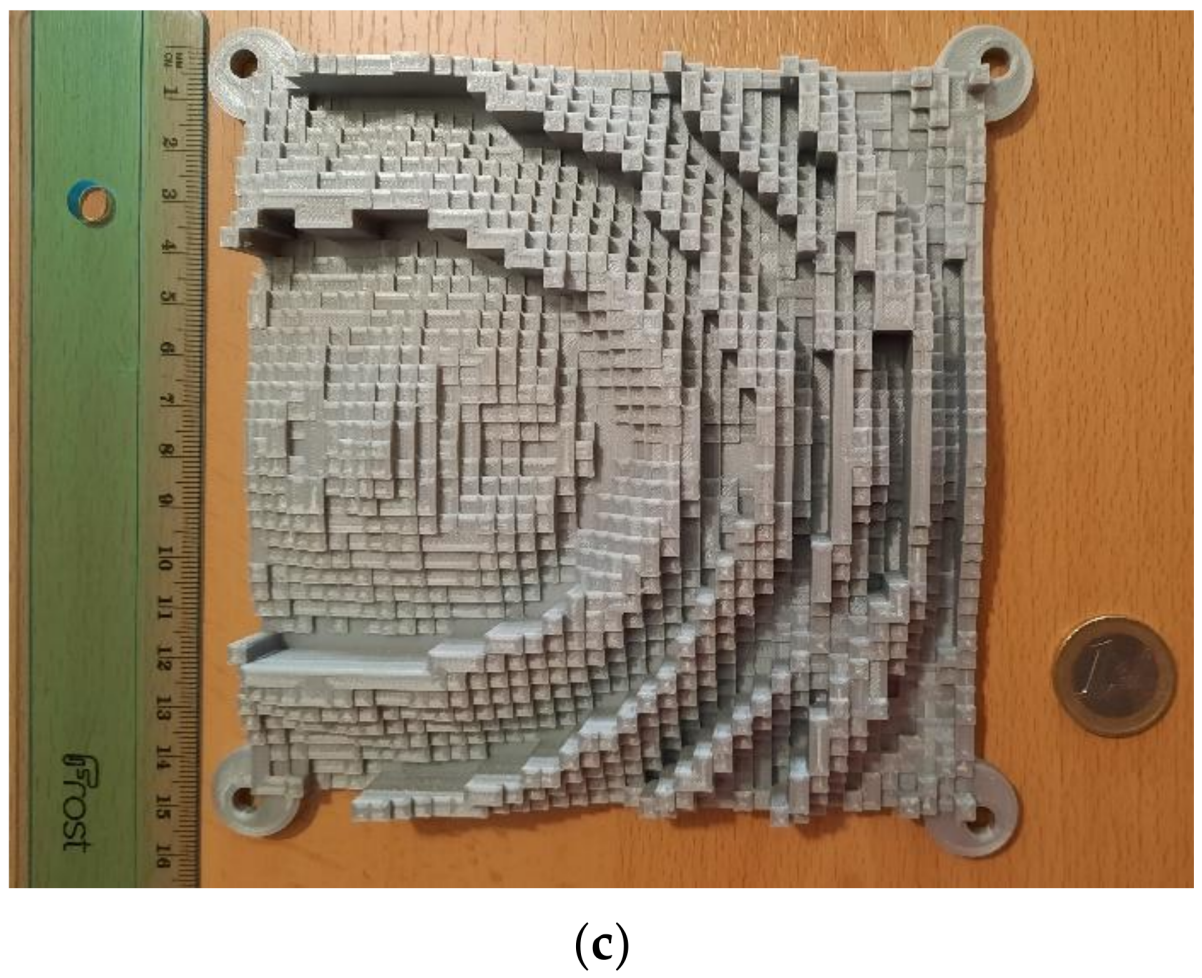
Case 1, dielectric TA: (**a**) required phase (0°–270°); (**b**) TA design; (**c**) fabricated TA.

Figure 10 shows the sequence of steps followed until the final prototype was reached. Firstly, it must be considered that, in order to minimize the shadowing effects, the elements with maximum height must be placed at the pointing direction [17]. Therefore, it may be necessary to add a phase constant to the original phase provided by the synthesis algorithm. Secondly, in order to have the TA as planar as possible, such that the implemented planar aperture model was suitable, the phase range was limited to 270° instead of 360°. Accordingly, Figure 10a shows the required phase at the TA once these modifications were implemented on $\phi_{TA}$. These processes slightly modified the results obtained for the NF distribution and FF pattern, but not significantly, as shown by the data included in last column of Table 1 for the dielectric TA. From this new phase distribution, the necessary slab heights were estimated for a polylactic acid (PLA) material with $\varepsilon_{r}=2.5$ and tgδ=0.005. These values are in good agreement with those provided in [26]. The resulting heights are shown in Figure 10b. These data were used in OpenScad [27], a free software for creating solid 3D CAD models, to generate the *stl* file to be used at the 3D printer. The printed TA is shown in Figure 10c. A ruler and a 1 EUR coin are included as a reference for size.

**Figure 11:**
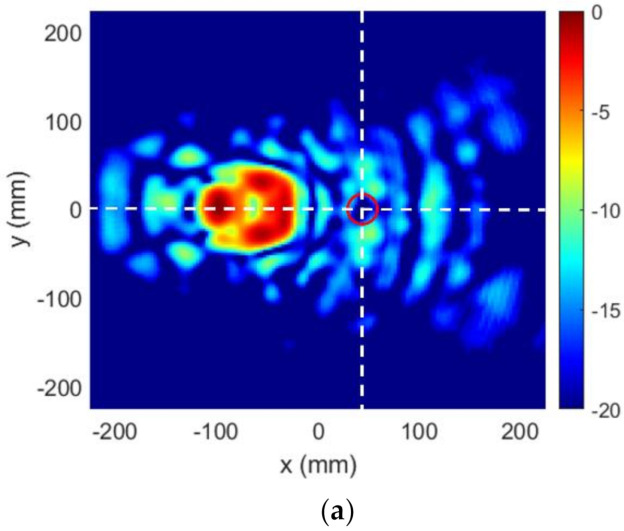

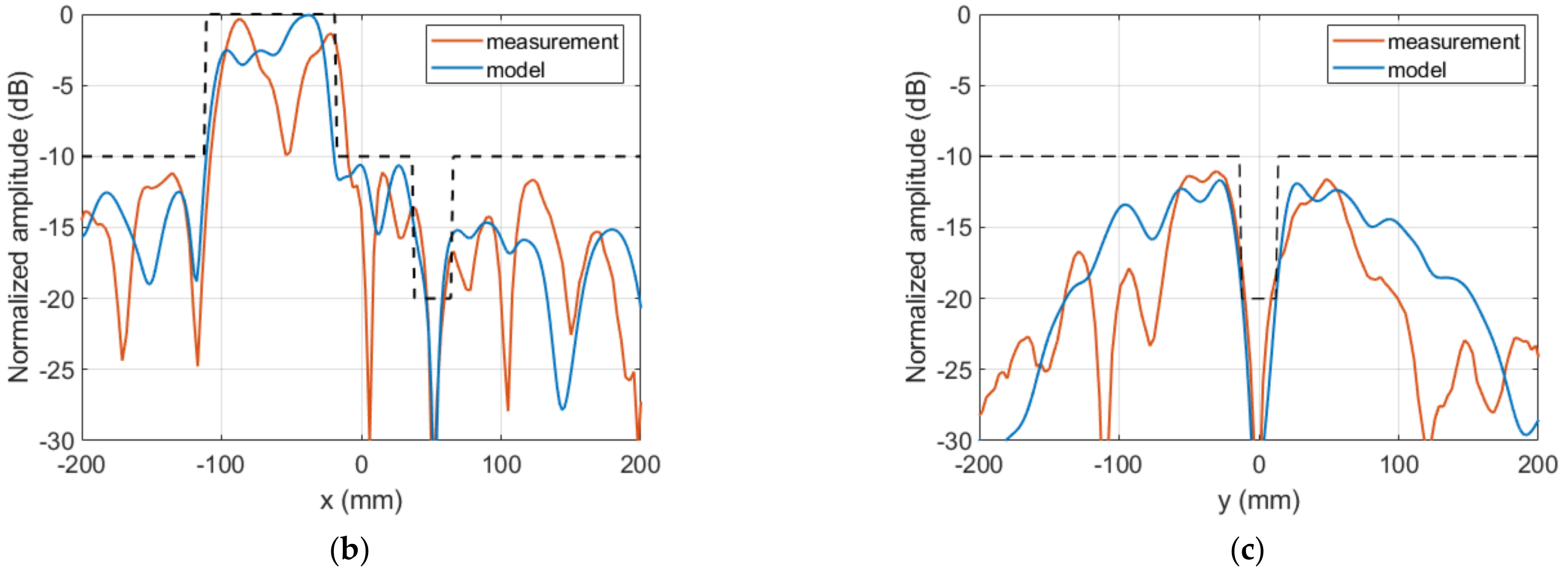
Case 1, NF results of the prototype. NF amplitude at z=D: (**a**) measurement (normalized amplitude, dB); (**b**,**c**) comparison of model result and measurement.

The NF distribution and the FF radiation pattern were measured in an anechoic planar range and an anechoic spherical range chamber, respectively. Both are facilities of the Signal Theory and Communications Research Group of the University of Oviedo. Figure 11a shows the near field measured for the manufactured TA, which can be compared with ${E^{\prime \prime }}_{NF}$ in Figure 7. A null can be appreciated at the desired location, highlighted with a red circle. Figure 11b,c compare the main cuts (white dashed lines in Figure 11a) of the measured NF amplitude with the results given by the model. The auxiliary templates used for the synthesis are included for comparison purposes. A good agreement is observed between the measurements and the model.

**Figure 12:**
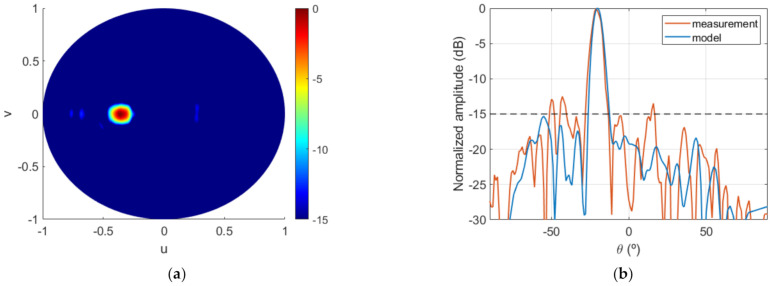
Case 1, FF results of the prototype: (**a**) measured radiation pattern (normalized amplitude, dB); (**b**) comparison of model result and measurement for cut v=0.

Lastly, Figure 12a shows the FF pattern measured at the anechoic chamber, and Figure 12b compares cut v=0 for model and measurement. As can be seen, the agreement between both results was good, and the manufactured prototype met the FF requirements satisfactorily. It must be said that the effect of the losses and the efficiency of the TA were not analyzed in this work. In that sense, the dielectric TA was mainly used as a proof of concept for the synthesis algorithm.

### 3.2. Case 2: Far-Field Beam and Near-Field Spot

The objective of this second case was to design a TA with the following constraints:

- FF: pointing at $\left(\theta =-20{}^{\circ},\;\phi =0{}^{\circ}\right)$ with SLL lower than −14 dB outside the main lobe.
- NF: spot at the position $\left[0.6D\cdot tg\theta^{\prime },0,\;0.6D\right]$ with $\theta^{\prime }=30{}^{\circ}$, spot width on the order of one wavelength, and level lower than −10 dB outside the spot.

**Figure 13:**
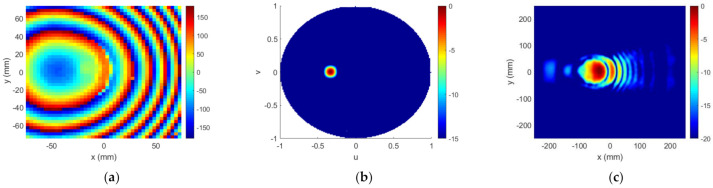
Case 2, outputs of Stage#1: (**a**) required phase ${\phi^{\prime }}_{TA}$ (°); (**b**) FF pattern $RP^{\prime }\left(u,v\right)$ (dB); (**c**) NF distribution ${E^{\prime }}_{NF}$ (dB) on plane z=0.6D.

A TA of 44×44 cells, i.e., D=152.3 mm at the operating frequency, was proven to be large enough to satisfy this objective, while the TA + spillover set consisted of 88×88 elements. The same steps as described for Case 1 were followed to achieve this objective and subsequently design and manufacture the dielectric TA. Figure 13 shows the results after Stage#1, where only FF requirements were considered. It can be seen in Figure 13b that the radiation pattern complies with the specifications. Moreover, the level was lower than −14 dB for 100% of the directions outside the main lobe, as shown in Table 2.

As explained in Section 2 and already performed for Case 1, the transmission phase ${\phi^{\prime }}_{TA}$ was used as input for Stage#2, where NF synthesis was performed to transform ${E^{\prime }}_{NF}$ in a new field distribution ${E^{\prime \prime }}_{NF}$ according to NF constraints. The NF grid consisted of 121,801 points (349 × 349) sampled every 0.15λ.

**Figure 14:**
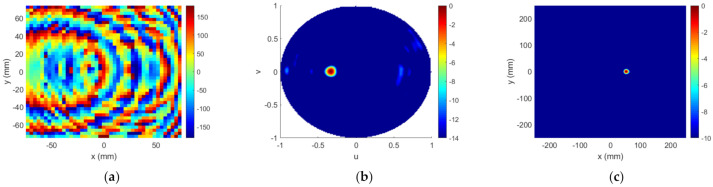
Case 2, outputs of Stage#2: (**a**) required phase ${\phi^{\prime \prime }}_{TA}$ (°); (**b**) FF pattern $RP^{\prime \prime }\left(u,v\right)$ (dB); (**c**) NF distribution ${E^{\prime \prime }}_{NF}$ (dB) on plane z=0.6D.

Figure 14a shows the necessary transmission phase ${\phi^{\prime \prime }}_{TA}$ to achieve the NF distribution ${E^{\prime \prime }}_{NF}$ seen in Figure 14c. The maximum field amplitude on the plane z=0.6D was found at $\left(x=51.92\;\mathrm{mm},\;y=0\right)$, and the spot width was $1.12\lambda \left(x\right)\times 1.04\lambda \left(y\right)$. Outside of the focusing spot, only 0.0123% of the positions presented a level above −10 dB.

**Figure 15:**
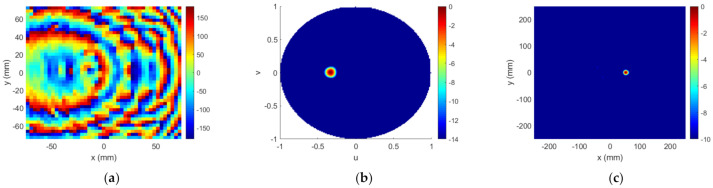
Case 2, outputs of Stage#3: (**a**) required phase $\phi_{TA}$ (°); (**b**) FF pattern $RP\left(u,v\right)$ (dB); (**c**) NF distribution $E_{NF}$ (dB).

**Figure 16:**
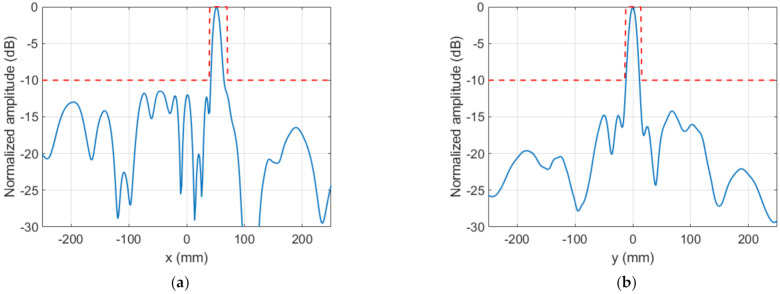
Case 2, Stage#3: main cuts of NF distribution ($E_{NF}$) and comparison with templates: (**a**) cut y=0; (**b**) cut x=51.92 mm.

Lastly, Stage#3 was executed, to try to correct the worsening of the FF results that occurred after Stage#2, and to reach a compromise between NF and FF results. A maximum of five iterations were run, and the best choice turned out to be the third one. Figure 15 illustrates the results after Stage#3, and Figure 16 shows the main cuts of NF distribution on the plane z=0.6D. All these results show that the algorithm successfully found a solution that satisfied both NF and FF requirements.

**Figure 17:**
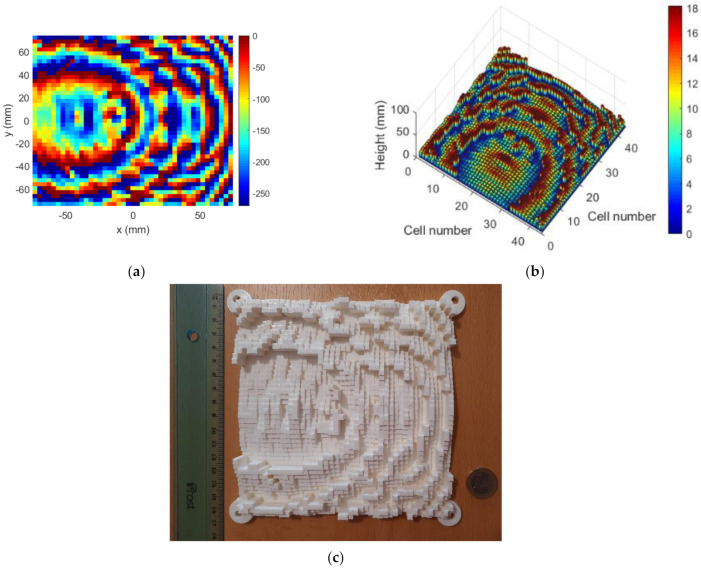
Case 2, dielectric TA: (**a**) required phase (0°–270°); (**b**) TA design; (**c**) fabricated TA.

The validation of the results was carried out following the same procedure as for Case 1. Then, from the result of Figure 15a, the dielectric TA was designed, previously adjusting the phase shift in the TA to be in the range of 0°–270°. The final design and the manufactured TA can be seen in Figure 17. Lastly, Table 2 summarizes the whole process for Case 2, in terms of noncompliance of the templates and execution times.

**Figure 18:**
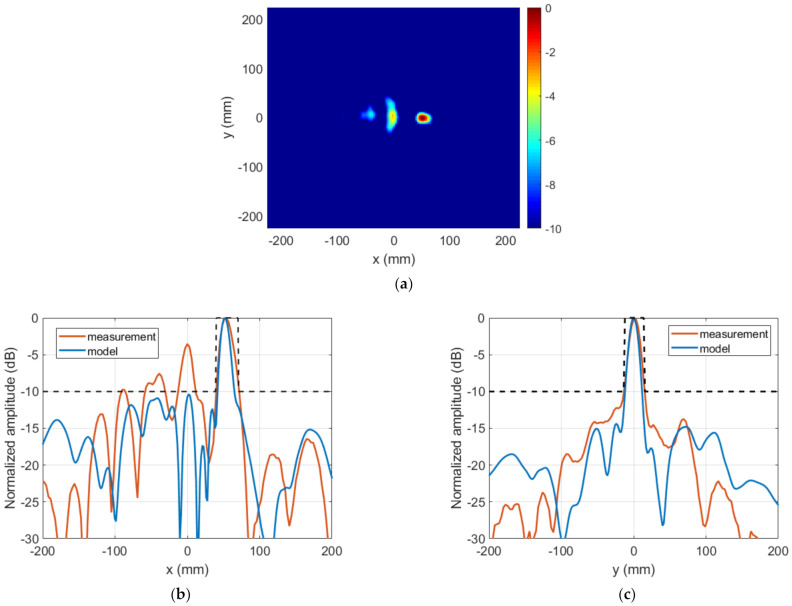
Case 2, NF results of the prototype. NF amplitude at z=0.6D: (**a**) measurement (normalized amplitude, dB); (**b**,**c**) comparison of model result and measurement.

**Figure 19:**
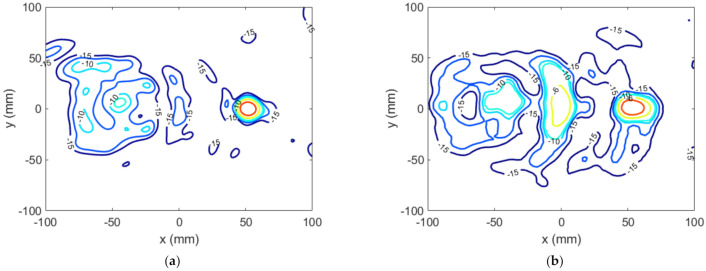
Case 2, NF normalized amplitude (dB) at z=0.6D around the focusing spot: (**a**) model result; (**b**) measurement.

The near field measured in the anechoic planar range for the manufactured TA is shown in Figure 18a, which can be compared with $E_{NF}$ in Figure 15c. The focusing spot was found at the desired location and with the desired width, as corroborated by Figure 18b,c. Outside the spot, the field level was under −10 dB as desired, except for the existence of some specific higher-level lobes. Figure 19 compares the simulated and measured NF distribution around the focal spot area. The result of the measure is coherent with that presented in [7], where some unexpected lobes also appeared when measuring a planar microstrip array synthesized to focus in two NF spots and radiate in a given FF direction.

**Figure 20:**
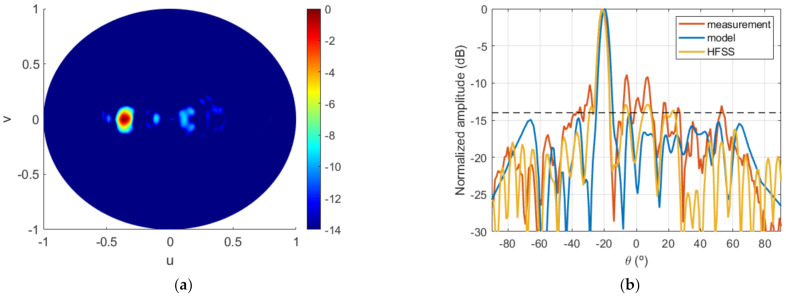
Case 2, FF results of the prototype: (**a**) measured radiation pattern (normalized amplitude, dB); (**b**) comparison of model result and measurement for cut v=0.

Lastly, the FF pattern measured at the anechoic chamber is shown in Figure 20a and compared with the model for cut v=0 in Figure 20b. The agreement with the results of the algorithm was good, and FF requirements were overall satisfactorily fulfilled, with the desired level being exceeded only at some specific directions. Moreover, the final system, i.e., feeding horn and dielectric TA of Figure 17c, was simulated with Ansys HFSS software [28] in order to have another element of comparison to contribute to the analysis and interpretation of the results. The result of this simulation is also included in Figure 20b. From the comparison between the three curves, it can be concluded that the HFSS result was intermediate between the model and the measurement. On one hand, the simulation showed good agreement with the model, thus validating the proposed algorithm. The small discrepancies were due to the simplifications assumed in the model [24]. On the other hand, the lobes that appeared in the measurement were intuited in the HFSS simulation, thus providing a better approximation to the measurements. This shows that a full-wave simulation could serve as a first validation of the synthesis technique that allows fine-tuning the TA design prior to fabrication.

The undesired spots or lobes in the measure could be related to the periodicity of the structure. A TA is, by definition, a quasi-periodic structure, which implies smooth transitions between cells. However, more demanding requirements render it more difficult to maintain this criterion. Accordingly, in this second case, transitions were to some degree less smooth than in Case 1, especially in certain areas of the TA. In the dielectric TA, this can generate shadowing effects of the taller slabs on their adjacent shorter cells. Since this effect was not considered in the algorithm, this could provide a plausible explanation for the appearance of those unexpected lobes in the measurement results. The effect of losses in the PLA is also an effect that should be studied in future work.

## 4. Conclusions

A technique was proposed for the synthesis of TAs when simultaneous NF and FF requirements are imposed. The aim was to have an efficient and fast tool; thus, the idea was to combine the tools previously proposed to separately perform NF and FF synthesis [21,22,23]. Then, the proposed algorithm successively performed an FF synthesis based on FFT, an NF synthesis, and a joint NF–FF synthesis. FF synthesis was very fast, even for a high number of iterations, since FFT requires extremely short computing times. For NF synthesis, the near field at each position was estimated as the sum of the independent FF contributions of each of the elements in the TA, and the synthesis of the TA phases was achieved through FFT, from the far field corresponding to the desired near field, i.e., once the near field was trimmed to the required template. The calculation of the near field required more computation time than the FFT. However, once FF synthesis was performed, only a few iterations were needed for NF synthesis. Lastly, NF–FF synthesis combined both procedures, seeking a compromise between FF and NF solutions, for which it required only a few iterations. The solution is neither optimal nor unique. However, the short computation time of only a few minutes makes it possible to run different cases rapidly and to study the best solution to the problem at hand. For example, different auxiliary masks or different TA sizes can be tested.

Two dielectric TAs were presented in this work as a proof of concept for the proposed algorithm. Previous work on FF-only [21,23] and NF-only [22] synthesis showed that, even though the algorithm is based on a planar aperture model [24], it can also be applied to the design of quasi-planar dielectric array lenses. The quasi-planar concept implies reducing the range of phase variation in the TA from 360° to the minimum possible value before the results of the algorithm are significantly worsened. It was proven in different studies [21,22] that this limit could be between 240° and 270°. This was again corroborated in this work, since the prototypes were designed and fabricated with a phase variation range of 270°. The measurements of the prototypes proved the effectiveness of the algorithm in meeting the specifications imposed for both the near and the far field, thus validating its results. As already noted in [7], a certain degradation of performance may occur when restrictions are imposed in both NF and FF without preventing the desired final result: a main lobe in the desired FF direction and a null or focal spot at the specified NF position. In addition, the objective of very short execution times was met. The two proposed cases can be run on a standard personal computer in about 10 min or less each.

In view of these results, the proposed approach can be a potential alternative to the search for solutions in complex scenarios with different coverage requirements in both far field and near field. Furthermore, dielectric TAs should be considered as an effective low-cost solution for antenna implementation. 

## Figures and Tables

**Table 1:** Case 1. Summary of synthesis results: errors and computing times.

		Stage#1	Stage#2	Stage#3	Dielectric TA
FF	% directions > −15 dB ^1^	0.0126%	0.0211%	0.0126%	0.0169%
	mean error (dB)	0.07	0.91	0.50	0.57
	variance (dB)	0.00	0.25	0.04	0.11
NF	% positions >−10 dB ^2^	--	0.3194%	0.4956%	0.4667%
	mean error (dB)	--	0.57	0.33	0.64
	variance (dB)	--	0.24	0.14	0.21
Number of iterations		100	5	5	--
Computing time	Workstation with Intel Core i7-9800X CPU @ 3.8 GHz	450 ms	70 s	70 s	--
	Laptop with Intel Core i5-5200U CPU @ 2.2 GHz	1.6 s	3 min 30 s	3 min 30 s	--

^1^ Outside of main beam. ^2^ Outside of main beam direction and null area, as defined by templates.

**Table 2:** Case 2: Summary of synthesis results: errors and computing times.

		Stage#1	Stage#2	Stage#3	Dielectric TA
FF	% directions > −15 dB ^1^	0.0000%	3.4196%	0.0970%	0.0801%
	mean error (dB)	--	1.21	0.13	0.23
	variance (dB)	--	0.78	0.01	0.02
NF	% positions > −10 dB ^2^	--	0.0123%	0.1913%	0.2044%
	mean error (dB)	--	0.21	0.40	0.76
	variance (dB)	--	0.01	0.11	0.39
Number of iterations		100	5	5	--
Computing time	Workstation with Intel Core i7-9800X CPU @ 3.8 GHz	450 ms	90 s	90 s	--
	Laptop with Intel Core i5-5200U CPU @ 2.2 GHz	1.6 s	5 min 10 s	5 min 10 s	--

^1^ Outside of main beam. ^2^ Outside of focusing spot.

## Data Availability

Not applicable.
